# Elucidating the Structural Features of ABCA1 in its Heterogeneous Membrane Environment

**DOI:** 10.3389/fmolb.2021.803078

**Published:** 2022-01-27

**Authors:** S. Sunidhi, Sukriti Sacher, Atul, Parth Garg, Arjun Ray

**Affiliations:** ^1^ Department of Computational Biology, Indraprastha Institute of Information Technology, Delhi, India; ^2^ Department of Computer Science, Indraprastha Institute of Information Technology, Delhi, India

**Keywords:** reverse cholesterol transport (RCT), ABCA1, ABC transporters, MDS (molecular dynamics simulation), membrane protein

## Abstract

ATP Binding Cassette Transporter A1 (ABCA1) plays an integral part in Reverse Cholesterol Transport (RCT) and is critical for maintaining lipid homeostasis. One theory of lipid efflux by the transporter (alternating access) proposes that ABCA1 harbours two different conformations that provide alternating access for lipid binding and release. This is followed by sequestration *via* a direct interaction between ABCA1 and its partner, ApoA1. The other theory (lateral access) proposes that ABCA1 obtains lipids laterally from the membrane to form a temporary extracellular “reservoir”. This reservoir contains an isolated lipid monolayer due to the net accumulation of lipids in the exofacial leaflet. Recently, a full-length Cryo-EM structure of this 2,261-residue transmembrane protein showed its discreetly folded domains and have detected the presence of a tunnel enclosed within the extracellular domains (ECDs) but not in the TMDs, giving it an outward-facing conformation. This structure was hypothesized to substantiate the lateral access theory. Utilizing long time-scale multiple replica atomistic molecular dynamics simulations (MDS), we simulated the structure in a large heterogeneous lipid environment and found that the protein undergoes several large conformational changes in its extremities. We observed that the cavity enclosed within ATP unbound form of ABCA1 is narrow at the distal ends of TMD as well as the ECD region substantiating the “lateral access” theory. We have also characterized ABCA1 and the lipid dynamics along with the protein-lipid interactions in the heterogeneous environment, providing novel insights into understanding ABCA1 conformation at an atomistic level.

## Introduction

ATP Binding Cassette transporters constitute a ubiquitous superfamily of integral membrane proteins that couple transport of a chemically diverse set of substrates across bilayers to hydrolysis of adenosine triphosphate (ATP). Amongst these, ABCA1 is involved in the transportation of lipids across the membrane (Phillips, 2018). ABCA1 plays a pivotal role in Reverse Cholesterol Transport (RCT) by facilitating the unidirectional net efflux of free cholesterol and phospholipids to poorly lipidated ApoA1 in plasma. This affiliation leads to the formation of nascent high density lipoprotein (HDL) (Ji et al., 2012). The physiological importance of ABCA1 is underscored by its association with various dyslipidaemic disorders such as familial HDL deficiency and Tangier disease, both of which are characterized by accumulation of cholesteryl esters in peripheral cells, particularly macrophages (Islam et al., 2018). Moreover, ABCA1-mutant mice show diminished Lecithin-cholesterol acyltransferase (LCAT) activity, lack of α-migrating HDL particles and triglyceride-rich pre-*β* HDL particles, highlighting its role in nascent HDL formation (Orsó et al., 2000; Aiello et al., 2002).

Although the role of ABCA1 in RCT has long been established, its functional characterization on the membrane is still in its elementary stages. It is reported that ABCA1 translocase activity leads to lipids reorganization such that free cholesterol in the membrane is more accessible to cholesterol oxidase. This reorganization is believed to be due to the partitioning of ABCA1 into non-raft domains caused by destabilization of rafts (Landry et al., 2006; Zarubica et al., 2009). In addition to ApoA1 dependent export of excess cholesterol, ABCA1 is also involved in flopping cholesterol from the inner to the outer leaflet of the plasma membrane (Okamoto et al., 2020). While several studies have biochemically characterized ABCA1 activity on the membrane and its involvement in nascent HDL generation, no work has been done to explicate ABCA1’s interaction with adjacent membrane lipids at an atomistic resolution.

**FIGURE 1:**
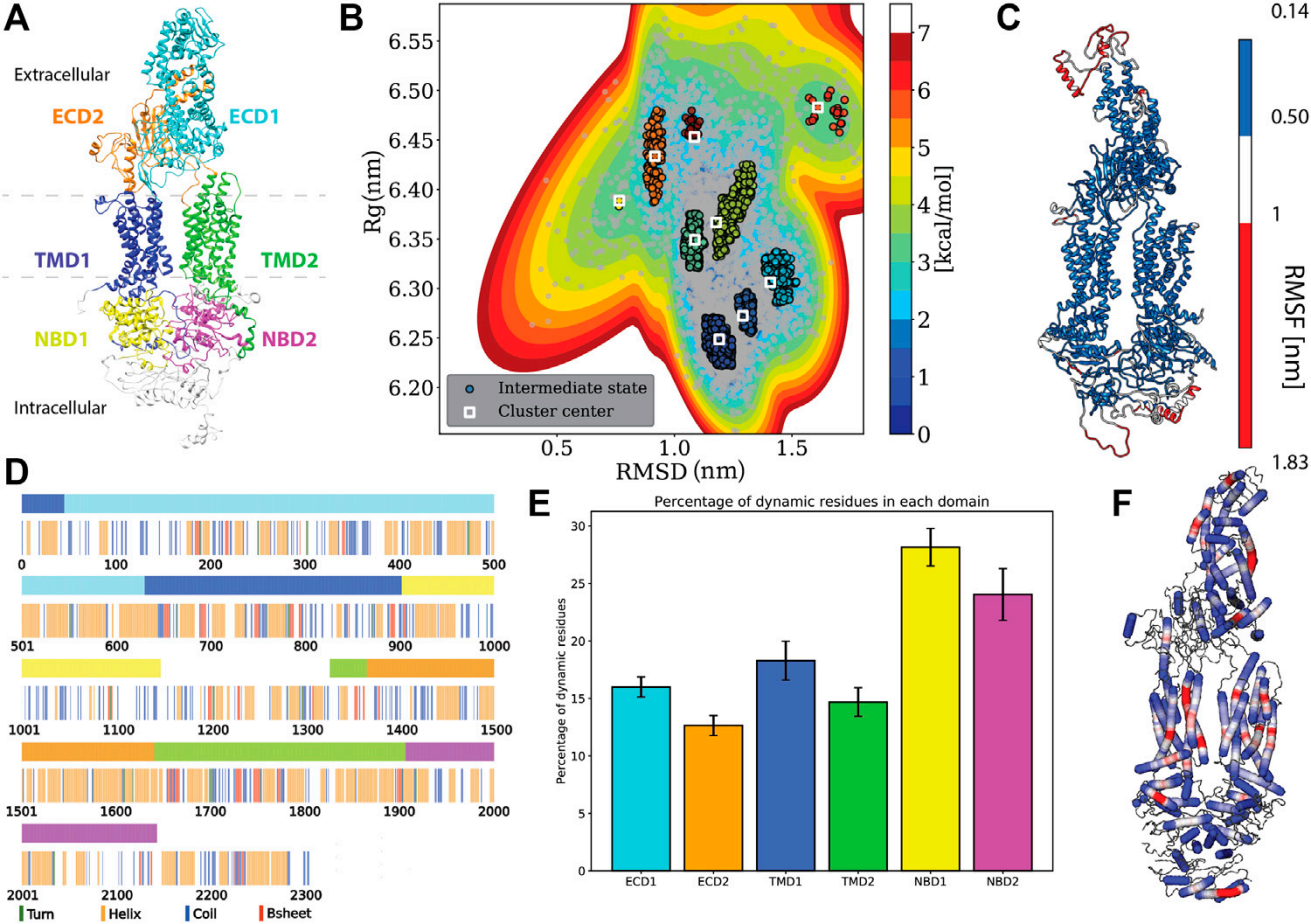
Protein specific analysis. **(A)** Domain assignment of ABCA1 (according to Qian et al., 2017) **(B)**. Free energy estimation and clustering showing distinct minima bins **(C)**. Root mean square fluctuation (RMSF) mapped onto the protein that showed the extremities of the protein being susceptible to large fluctuations. **(D)** Residue-wise and domain-wise assignment of favoured secondary structure amongst turn (green), helix (orange), coil (blue), b-strands (red) and dynamic residues (white) according to the **(A)** colour scheme (top bar) **(E)** Percentage of dynamic residues in each domain, where the color of each bar corresponds to the domains in**(A)**. **(F)** Degree of flexibility of helices constituting the protein where regions in red indicate areas of highest helix axis angle (>15°) and flexibility, white for intermediate angle magnitudes (0–15°) and blue for angles close to, or at 0°.

ABCA1 is a lipid transporter protein made up of 2,261 residues that form six domains: two transmembrane domains (TMDs, consisting of six membrane-spanning helices each); two nucleotide binding domains (NBDs) in the cytoplasm that serve to couple ATP hydrolysis to translocase activity; and two large extracellular domains (ECDs) that are implicated in protein-protein interactions as well as regulatory roles (Qian et al., 2017) (Figure 1A). The transporter activity of the members of the ABC family follow the mechanism of “alternating access”. According to this, the NBDs form a tight dimer with two molecules of ATP on their binding, leading to a change in the conformation of TMD from inward-facing to outward-facing. This forms a “channel” for the transport of substrate molecules (Phillips, 2018). However, the recent Cryo-EM crystal structure (PDB ID: 5XJY) of a nucleotide-free ABCA1 unveiled a snapshot of the protein such that the TMDs faced outwards (pore opening towards the extracellular milieu), making ABCA1 an outlier amongst proteins of the ABC family whose structures have been captured (Qian et al., 2017). Moreover, this structure did not observe a continuous central cavity in the TMD region. Additionally, this ABCA1 structure also revealed a polar cluster on one side of TMD1. This pocket was speculated to bind polar head groups of lipids and facilitate their flopping movement across the hydrophobic barrier. This structural feature suggested a lateral mechanism for substrate entrance (from the membrane inner leaflet), which is in contrast with the conventional paradigm of alternating access.

The structure also noted that the two extended ECDs adopt a novel fold. ECD1 folds to form four distinct regions: a base domain, helical domain 1 and 2, and a lid domain, while ECD2 packs tightly against it (Qian et al., 2017). The ECDs of ABCA1 enclose a predominantly hydrophobic tunnel ∼ 60 Å in height that is open to the extracellular milieu on both its distal and proximal ends. This hollow interior is conjectured to serve as a temporary storage or delivery passage for lipids (Qian et al., 2017; Ishigami et al., 2018). The Cryo-EM structure showed that the distal end of ECD1, close to the lid, forms a narrow opening that precludes the passage of lipids. The proximal end is, however wide enough for the lipids to pass. Therefore, this region is proposed to be the site of delivery of lipids to ApoA1, which is postulated to associate with the transmembrane protein laterally. Additionally, the proximal tunnel opening is ∼30 Å away from the upper end of the transmembrane cavity. Therefore, a pronounced conformational change (in response to ATP hydrolysis) is predicted to occur in order to facilitate the delivery of lipids from the membrane to ECDs (Nagao et al., 2012; Qian et al., 2017).

In this study, we have employed all-atom molecular dynamics to simulate the ATP unbound ABCA1 monomer in an explicit heterogeneous membrane bilayer to refine the original model. Additionally, we have tried to assess the evidence ascertained in the Cryo-EM structure and its proposed evidence towards “lateral access theory”. We highlight the effect of immersing ABCA1 in its natural habitat on its overall architecture, volumetry as well as its secondary structural makeup. We have also quantitated the protein-lipid interactions in order to elucidate the effect of the presence of protein on membrane dynamics. This study sheds light on the protein-lipid interactions that impact ABCA1 stability in the membrane.

## Methods

### Structure Preparation and Classical Molecular Dynamics

Cryo-EM structure of ABCA1 with an overall resolution of 4.10 Å (PDB ID: 5XJY) was modelled for missing regions using Modeller (Sali and Blundell, 1993) and a heterogeneous membrane was assembled around the protein using replacement method in CHARMM-GUI. The method involved introduction and distribution of lipid-like pseudo atoms around the protein and then their replacement by chosen lipid molecules, consecutively (Jo et al., 2009). The assembled heterogeneous membrane was made of 1-palmitoyl-2-oleoyl-sn-glycero-3-phosphocholine (POPC) (482 molecules), palmitoyl sphingomyelin (PSM) (108 molecules), Cholesterol (CHL) (48 molecules), 1-palmitoyl-2-oleoyl-sn-glycero-3-phosphoinositol (POPI) (36 molecules), 1-Palmitoyl-2-oleoyl-sn-glycero-3-phosphoethanolamine (POPE) (30 molecules) and 1,2-palmitoyl-oleoyl-sn-glycero-3-phosphoserine (POPS) (24 molecules), Lysophosphatidylcholine (LLPC) (18 molecules) (Duong et al., 2006; Maxfield and van Meer, 2010; Lund-Katz et al., 2013; Casares et al., 2019).

MD simulations were performed with the GROMACS 4.6.1 (Bekker et al., 1993; Hess et al., 1998; Lindahl et al., 2001). The initial system was built using CHARMM-GUI and is represented by CHARMM36 all-atom force-field (Brooks et al., 2009; Denning et al., 2011; Huang and MacKerell, 2013). The water was modelled using the TIP3P representation (Jorgensen et al., 1983). Each of the five starting conformations were placed in a dodecahedral water box (volume = 6,477.957 nm^3^) large enough to contain the protein-membrane complex and at least 1.0 nm of solvent on all sides. Periodic boundary conditions were used, and the long-range electrostatic interactions were treated with the particle mesh Ewald method (Darden et al., 1993) using a grid spacing of 0.12 nm combined with a fourth-order B-spline interpolation to compute the potential and forces in-between grid points. The real space cutoff distance was set to 1.2 nm and the Van der Waals cutoff to 1.2 nm. The bond lengths were fixed (Hess et al., 1998), and a time step of 2 fs for numerical integration of the equations of motion was used. Coordinates were saved every 200 ps. The pressure coupling was done by employing a Parrinello-Rahman barostat (Parrinello and Rahman, 1981) using a 1 bar reference pressure and a time constant of 5.0 ps with the compressibility of 4.5e-5 bar using a semi-isotropic scaling scheme. Eighty-two positive counter-ions (K+) were added, replacing eighty-two water molecules to produce a neutral simulation box. All the starting structures were subjected to a minimization protocol for 5,000 steps using the steepest descent method followed by six sequential equilibration steps with restraints on the protein atoms as per the CHARMM-GUI protocol.

Five independent trajectories, each of 1 μs at 300 K were carried out. The combined time-scale of our simulations is 5 μs

A control membrane (without the protein) with same composition (POPC: 482, PSM: 102, CHL: 48, POPI: 36, POPE: 30, POPS: 24, LLPC: 18) was similarly constructed using CHARMM-GUI using the CHARMM36 force field. One hundred and forty-one positive counter-ions (K+) as well as eighty-one negative counter-ions (Cl-) were added, replacing two hundred and two water molecules to produce a neutral simulation system. This system was subjected to minimization and equilibration as previously mentioned, in accordance with CHARMM-GUI protocol. The control membrane was simulated for 500 ns using similar conditions and with similar parameters as that of the protein-lipid complex. The details of the simulations are given in Table 1.

**TABLE 1:** Simulation details of the study.

System	Time	Total size (atoms)	# Water	# POPC	# PSM	# CHL	# POPI	# POPE	# POPS	# LLPC
ABCA1 in membrane	1 μs (five replicates)	664,552	177,349	482	108	48	36	30	24	18
Control membrane	500 ns	188,218	30,678	482	108	48	36	30	24	18

### Simulation Analysis

**Domain assignment:** The six domains in the ABCA1 structure were assigned and colored accordingly using UCSF Chimera 1.14 (Pettersen et al., 2004). The domains were assigned in accordance with the structure of the human lipid exporter ABCA1(Qian et al., 2017). The range of residues and the assigned color(s) are as follows: ECD1 (46–630, cyan), ECD2 (1366–1640, orange), TMD1 (1–45 and 631 to 902, blue), TMD2 (1327–1365 and 1641 to 1906, lime), NBD1 (903–1147, yellow), and NBD2 (1907–2143, magenta).

**Domain motion analysis:** Principal component analysis was performed using the covariance matrix of the atomic coordinates throughout the simulation. Eigenvectors (representing modes of fluctuation) and eigenvalues were obtained by diagonalizing this matrix. Eigenvector 1, representing the most dominant mode of motion, was used to produce a PDB file containing 100 conformations along this vector. This was visualized in the Normal Mode Wizard of VMD (Humphrey et al., 1996).

**Free energy estimation and clustering:** InfleCS was used to estimate the density and free energy using Gaussian mixture models (GMM) (Westerlund and Delemotte, 2019). A 2D array of RMSD and Rg of all five sims was given as the input. The estimated density was then clustered using InfleCS clustering and the free energy was visualised. The core states were identified at density maxima using the estimated Gaussian mixture density and the points were divided into clusters.

**Helix flexibility:** VMD Bendix plugin (Dahl et al., 2012) was used to calculate and visualize both dynamic and static helix geometry. RWB or Red-White-Blue heatmap colors were used for visualization. Red indicates the areas of the highest helix axis angle (>15°), followed by white for intermediate angle magnitudes (0–15°) and blue for angles close to, or at 0⁰. Therefore, red-colored regions are highly flexible while blue regions are rigid. The Bendices ends that are at an angle of 0 per definition are not resolved by the axis-generating algorithm.

**Average lipid thickness**: Lipid thickness across both leaflets was calculated after equilibration using the MEMBPLUGIN (Guixà-González et al., 2014) software. MEMBPLUGIN was used for calculating average membrane properties such as the membrane thickness. The Membrane Thickness tool contained in this software calculates the average membrane thickness over a chosen trajectory by measuring the distance between the two density peaks of user-selected atoms (phosphorus) belonging to the head group of phospholipids. The average lipid thickness was calculated for all five sims post stabilization. The mean average thickness across all five simulations was then plotted.

**Membrane partial density:** This metric was calculated using g_mydensity on a grid with a spacing of about 0.2 nm (Castillo et al., 2013). Therefore, each element of volume (voxel) was ∼ 0.2 nm × 0.2 nm × *Z* nm, where *Z* is the dimension of the box in the direction of the membrane normal. Partial densities were calculated as the average mass of the particles present in the voxel divided by the volume of the voxel; only lipids were taken into account.

**Thickness maps:** Lipid thickness maps for both leaflets were generated *via* MEMBPLUGIN (Guixà-González et al., 2014) software. Time-averaged deformation maps were generated by extrapolating the *z* coordinates of P atoms into a regular orthogonal grid in the *XY* plane. Irrespective of the leaflet, deformation is positive when it expands the membrane, and negative otherwise. The thickness maps were visualized in a contour plot.

**Lipid clustering**: Cluster analysis was done using an in-house Python script. The last frame of the simulated trajectory was extracted in the PDB format. The PDB file thus generated was parsed to extract all the relevant information for all the atoms in the system. For each lipid/protein residue, a mean position was calculated by taking the arithmetic mean of the *x*, *y*, and *z* coordinates of the atoms pertaining to the lipid/protein residue in question. Taking the mean *z* coordinate of the thus generated coordinates, the membrane bilayer was split into two monolayers, i.e. the top and the bottom layer. The desired layer was then further divided into square sections of 6 Å length. For each such box, the number of residues of the desired lipid class lying inside the box was calculated and plotted. At the same time, the protein residues lying inside the desired monolayer were calculated using the *z* coordinates as an indicator of the same. For all the protein residues thus identified, a similar estimation of a single coordinate was done similar to the way it was done for lipid residues. Finally, all the little 6 Å boxes containing at least one such protein residue were marked on the plot with a blue square, while the lipid residues were marked with colors according to their prevalence (frequency).

**Bilayer curvature**: To check whether the lipid membrane was curved to any extent because of the presence of the protein and to calculate the mean curvature of a membrane leaflet, the g_lomepro suite (Gapsys et al., 2013) was used. To determine the mean curvature, the lipid coordinates along the bilayer normal were first mapped onto a grid space, using a modified version of the GridMAT-MD algorithm. Subsequently, a filter function was used to transform the grid-mapped coordinates to Fourier space and was then recovered *via* an inverse transform. First- and second-order derivatives of the filtered coordinates were used to calculate the mean curvature. In the current study, the ABCA1 protein was centred, and the last frame of each trajectory was analyzed. The mean curvature was individually analyzed for each set. The phosphate head groups were used to represent the membrane. A leaflet is defined to be positively curved if it bends away from the bilayer centre toward ABCA1 and negatively curved if it bends toward the bilayer centre away from ABCA1. Further, the visualization of the curved structures was done using VMD (Humphrey et al., 1996) by keeping bin-x and bin-y parameters as 100.

## Results and Discussion

### Stable Conformation of ABCA1 in a Heterogeneous Bilayer

Using multiple long scale (five replicates of 1 μs each) all-atom classical molecular dynamics, we established the stable conformation of the protein in a heterogeneous lipid membrane. The deviation from the initial Cryo-EM structure is shown in Supplementary Figures S1A,B that depicts the mean RMSD and Rg values of the five replicate simulations. It was observed that the final structure from the simulated trajectory stabilized with ∼1.2 nm deviation on average from the initial structure. This stable structure showed an increase of 0.17 nm in its Rg value. Next, we clustered the various protein conformations that existed throughout our simulations based on the structural deviations that it underwent from its starting Cryo-EM state. Structural deviations characterized by the RMSD and Rg values yielded seven distinct clusters (Figure 1B). We visualized a representative structure from each of these energy bins and found that ABCA1 underwent drastic changes in the ECD domain (Supplementary Figure S2).

Additionally, the analysis of the mean structural fluctuations of protein throughout the simulation length also revealed higher fluctuations (>1 nm) in the extremities, notably in the ECD1, NBD1 and NBD2 (Figure 1C; Supplementary Figure S3) while the major portion of the protein remained relatively stable throughout the simulation. The domain concerted motion and PCA analysis of the trajectories also depict maximum movement in these regions (Supplementary Figure S4). This result is expected since both these regions are water-exposed and are known to undergo conformational changes during the ATP hydrolysis cycle (Oliveira et al., 2011; Nagao et al., 2012).

We also characterized the tunnel enclosed within the protein Garg et al., 2020. The overall conformation as well as the volumetry of the tunnel within the TMDs is suggestive of the “outward-facing” conformation of ABCA1 in its nucleotide-free state where the two TMDs are seen contacting each other close to the inner leaflet (Supplementary Figure S5A). Four out of five of our simulations showed a significant narrowing in this region with the volume being too small to accommodate a single lipid (Greenwood et al., 2006). The outward-facing conformation, named so, to depict that the pore opening lies towards the extracellular-milieu represents the substrate-bound state of the ABC transporter achieved after ATP hydrolysis. Therefore, ABCA1 being in this conformation in its native state is indeed unexpected. Qian et al. argued that the lipid substrates from the inner leaflet may still be able to access the substrate-binding site, considering all the other side surfaces of helices making up TMD1 and TMD2 were found to be completely exposed to lipids. However, our simulations were not suggestive of any probable lipid-binding site.

Similarly, we also characterized the hydrophobic cavity inside the ECDs and observed that in the nucleotide unbound state, the distal end of the ECD cavity, close to the lid, precludes lipid efflux. Despite the structural fluctuations in the extremities of ECD, the volume of this region was observed to be too low to harbour even a single lipid (Supplementary Figure S5B). Although it can be speculated that a conformational change in the ECDs due to ATP hydrolysis may lead to significant widening in this region.

### Secondary Structural Characteristics of Adenosine Triphosphate Unbound ABCA1 Monomer in Lipid Bilayer

We utilized all the five replicates to elucidate the secondary structure assignment for each residue. In order to do this, the probability of every residue to conform to a particular secondary structural element was computed (Supplementary Figure S6). Based on a residue’s propensity to exist as either a helix, turn, strand or coil, for 70% of the time throughout the simulation, a secondary structure was assigned to it (Figure 1D). It was observed that the protein structure was predominantly constituted by helices (41% residues), followed by coil (21% residues), while turns and *β*-Strands were least favoured (14.34 and 5.41% respectively) (Figure 1D). Similarly, we also computed the secondary structure makeup for each domain and observed that all six domains are majorly constituted of helices followed by coils (Supplementary Figure S7). Additionaly, we also quantified those residues that did not conform to any particular secondary structure throughout our simulation (existed as a secondary structure for <70% of times throughout simulation). The percentage of these dynamical residues that make up each of the functional domains of the protein are shown in Figure 1E. These residues, displaying fast dynamics for inter-change of secondary structures, were found to predominantly exist in NBDs highlighting the underlying conformational flexibility of this region.

Additionally, it was observed that most of the helices that made up the protein were rigid (blue, helix angle close to 0), while a few of them displayed a fair degree of bending (red) (Figure 1F). Incidentally, most of the helices showing flexibility lay within the TMDs. Helix flexibility has been shown to play a role in mechanisms of gating in ion channels (Tieleman et al., 2001). Since ABCA1 obtains its substrate laterally from the membrane, the helical flexibility of this region may enable the substrate uptake mechanism.

### Influence of ABCA1 on Membrane Curvature

In order to understand the fate of lipids in the heterogeneous membrane due to the presence of ABCA1, we simulated another heterogeneous membrane with the same composition (see Materials and Methods for details) to serve as our experimental control. The average membrane thickness (of outer as well as inner leaflet) for both the membranes was computed. Our results indicated the membrane thickness to be fairly constant throughout all the five simulations as well as control membrane (Supplementary Figure S8). The average thickness of the bilayer ranged from 41.2–41.9 Å for the membrane which had ABCA1 embedded in it, while that of the control membrane ranged from 41.7–42.6 Å.

**FIGURE 2:**
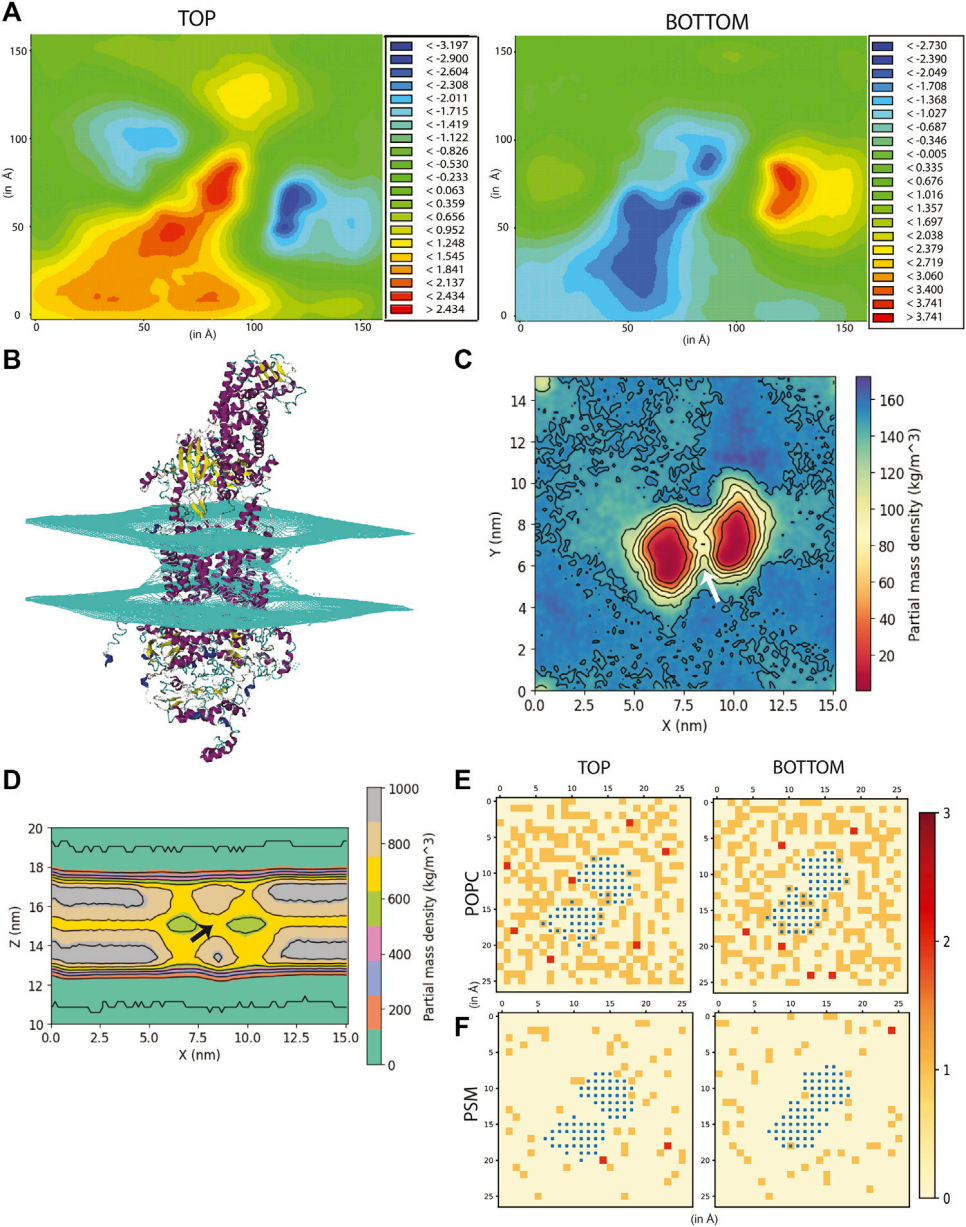
Effect of ABCA1 on membrane curvature and dynamics. **(A)** Heat maps depicting average membrane thickness of the top and bottom leaflet of the membrane throughout simulation. **(B)** Curvature induced in the membrane due to presence of protein. Partial density of lipids across two leaflets along **(C)** *XY*-plane and **(D)**. *XZ*-plane. Arrow [colored white and black in **(C,D)** respectively] depicts the island of lipids within the TMDs. Lipid clustering showing the frequency of lipids (shown as red, orange and cream) of **(E)**. POPC and **(F)**. PSM around protein (colored blue). The membrane is divided into square box of edge 6 Å each and frequency in each of these boxes has been computed and depicted in the figure.

Figure 2A depicts the time-averaged deformation map for the top (average thickness ranged from −3.19 to 2.43 Å) as well as the bottom leaflet (average thickness ranged from −2.73 to 3.74 Å). This deformation map revealed that the outer leaflet experienced a positive deformation at the centre (the region in red having thickness 1.24 to 2.43 Å) while the lower leaflet underwent a negative deformation (the region in blue -0.34 to −2.73 Å) compared to the control membrane (Supplementary Figure S9). These striking differences in membrane thickness imply that the presence of ABCA1 leads the outer leaflet to be expanded while the lower leaflet condenses at the site of implantation of protein in the bilayer. Next, to ascertain whether any distortion was present in the membrane, mean curvature of both the leaflets along the membrane normal was calculated using a modified GridMAT algorithm. We observed that both the leaflets were found to be curved towards the middle of the bilayer at the points where the TMDs intersected with the bilayer (Figure 2B). This indicates that the presence of protein indeed, induces curvature in the bilayer. Interestingly, ABCA1 activity is believed to result in condensation of outer leaflet and expansion of the inner leaflet, which is in complete contrast with our observation of its resting-state conformation (Vedhachalam et al., 2007). It is likely that this compression of the bilayer is necessary for lipid translocation (Vedhachalam et al., 2007).

The partial density profile of the simulation along *XY*-plane showed an overall reduction in lipids (normalized across the two leaflets) around the protein (Figure 2C). The contours in the density profile computed along the *XZ*-plane) too showed that the membrane curves at the centre where the TMDs of ABCA1 are rooted (Figure 2D; Supplementary Figure S10). Interestingly, there exists an island of lipids within the region between the two TMDs of the protein (Figures 2C,D). This region may serve as the source of substrate for the protein.

### Influence of ABCA1 on Lipid Dynamics

Next, the average area spanned by the heterogeneous membrane with protein as well control membrane was computed as a measure of their stability throughout the simulation (Supplementary Figure S11). It can be observed that the average area spanned by the membrane with ABCA1 was a little higher than the control membrane (60.8–61.9 Å^2^ and 55.0–57.5 Å^2^ respectively).

**FIGURE 3:**
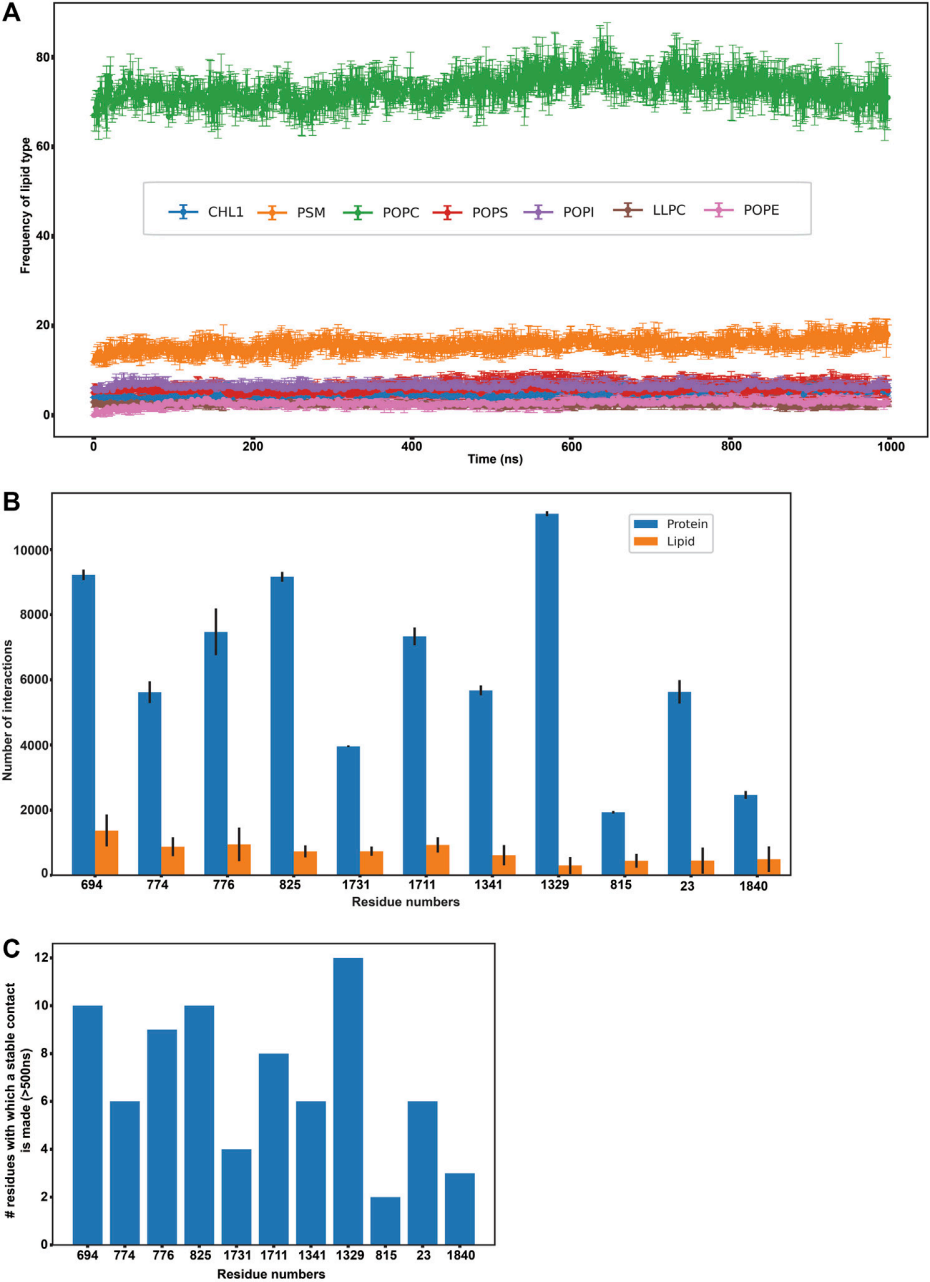
Protein-lipid interactions’ analysis. **(A)** Frequency of each lipid type surrounding the protein residues within a radius of 0.5 nm throughout our simulation **(B)** Frequency of protein-protein and protein-lipid interactions of the eleven disease-related protein residues **(C)** Number of stable protein-protein contacts made by the eleven disease-related protein residues (>500 ns of simulation time).

In order to further explicate the protein-lipid interactions at play, we performed clustering of each lipid type to generate separate heat maps based on lipid counts around the protein. Supplementary Figures S12, S13 depicts the spatial location of each of the lipid types around the protein. It was observed that the most abundant lipid around the protein residues in both the top and bottom leaflet was POPC followed by PSM. Figures 2E,F illustrate the heatmap of POPC and PSM respectively, where the gradient colours represent the number of lipids (POPC or PSM) molecules in the cluster and the blue squares represent the protein residues. While both the lipids did not appear to cluster as such, both POPC and PSM appeared to form a shell around the protein residues that were immersed in the membrane. We also determined the different lipid types that surrounded the entire protein within a radius of 0.5 nm throughout the simulation (Figure 3A). We assumed that these lipid residues may be interacting with the corresponding protein residues owing to their close vicinity. Our results indicated that the most prevalent lipid type within the shell radius of the protein is POPC followed by PSM.

### Elucidating the Protein-Lipid Interactions That Stabilize ABCA1 in the Membrane

In order to study the protein-lipid interactions, we focussed on the protein residues that were found to interact with these surrounding membrane lipids for more than 500 ns throughout the simulation. A total of 156 such residues were found and the total number of interactions for each of these residues was quantified (Supplementary Figure S14). It was observed that some of these residues did not interact with any lipid residues initially (depicted in dark blue at the bottom of the heatmap), but were found to form transient interactions with 1-4 lipids as the course of simulation progressed. All such protein-lipid interactions were aggregated and have been depicted according to lipid types that they interact with in Supplementary Figure S15.

Subsequently, we analyzed if any of these 156 residues (that were found to actively interact with lipids throughout our simulation) were implicated in any of the ABCA1 related disorders in order to extrapolate the importance of their interactions with surrounding lipids in protein stability or function. We mined ClinVar to obtain all the non-synonymous mutations in ABCA1 reported to date. All those residues that were reported to be pathogenic or disease-related in ClinVar and were interacting with lipids for >500 ns in our simulation were analyzed. Furthermore, SNPs and Go (Capriotti et al., 2013) were also used to ascertain the disease association of the shortlisted nsSNPs. SNPs and Go results include, in addition to itself, predictions from PhD-SNP (Capriotti and Fariselli, 2017) and PANTHER (Tang and Thomas, 2016). We observed that 11 amongst these 156 residues (C23Y, L694del, T774P, K776N, E815G, V825I, F1329V, R1341T, V1711I, S1731C, F1840fs) were associated with ABCA1 related disease (Supplementary Table S1). These were associated with pathogenic phenotypes such as increased cardiovascular risk and plasma cholesterol levels. Figure 3B enumerates the number of protein-protein as well as protein-lipid interactions that these 11 residues participate in while Figure 3C depicts the intensity of protein-protein contacts of these 11 residues. Additionally, it was also observed that these residues made contact with both the outer and the inner leaflet. They were also found to interact with POPC and PSM mostly (Supplementary Figure S15). Our results are suggestive of the role of these residues in several stabilizing interactions (both protein-protein as well as protein-lipid in nature), which is why mutations in these regions affect protein function and are potentially destabilizing in nature.

## Conclusion

The results presented herein represent the first simulation of an ATP unbound monomeric ABCA1 in a heterogeneous membrane environment. Though the simulation stabilized structure obtained showed a large deviation (∼1.4 nm) from the Cryo-EM structure, the most dynamical fluctuations were observed in the ECD throughout our simulations. The overall architecture, as well as volumetry of the tunnel enclosed within ABCA1, is indicative of its outward-facing conformation. Despite the narrowing at the cytosolic end of TMD due to contacts made by TMD1 and TMD2, our results also depicted that the side surfaces of TMDs are completely immersed by lipids and are in agreement with previous literature. These observations are suggestive of a lateral access mechanism of substrate entrance, such that the lipid enters the TMD tunnel through the side surfaces of TMDs (Qian et al., 2017).

Additionally, the volume profile of the distal end of the ECD tunnel is suggestive of its role as a temporary storage site as opposed to an exit point. However, considering the large fluctuations in the extremities of ECD1 (lid domain), it is plausible that a conformational change upon lipid binding and ATP hydrolysis may push the two ECDs apart, making way for lipid egress.

We have also documented the protein-lipid interactions that detail the effect of protein on membrane dynamics at an atomistic resolution. Our observations suggest that the presence of ABCA1 exerts a differential role on leaflet curvature, due to which both the leaflets curve inwards at the site of implantation of the protein. The lipids were also found to be sparsely packed around the protein (decreased overall density), when the protein was in its inactive state (ATP unbound).

Recently, reverse cholesterol transport has surfaced as a focal point for the regulation of lipid metabolism, especially given its role in cardiovascular disease. Since ABCA1 mediated lipid efflux is the first step of the pathway, establishing its stable conformation and mechanism of action is of immense importance. In summary, our study has refined the original model of the ATP unbound ABCA1 by studying it in its native environment and these novel findings will pave the way for future studies.

## Data Availability

Publicly available datasets were analyzed in this study. This data can be found here: RSCB PDB ID:5xjy. The following is the link used for PDB ID 5xjy available on RCSB used in our study. https://www.rcsb.org/structure/5xjy
