# Supplementary material for: Elucidating the Structural Features of ABCA1 in its Heterogeneous Membrane Environment
Source: Front Mol Biosci. 2022 Jan 27;8:803078. doi: 10.3389/fmolb.2021.803078 (PMC8830745; doi:10.3389/fmolb.2021.803078)
Supplement: Supplementary file 1 [file DataSheet1.pdf]

Elucidating the Structural Features of ABCA1 in Heterogeneous Membrane Environment - Supplementary Data

S. Sunidhi [1]†, Sukriti Sacher [1]†, Atul [2], Parth Garg [1], Arjun Ray [1] *

[1]Department of Computational Biology, Indraprastha Institute of Information Technology, Delhi, New Delhi, India

[2]Department of Computer Science, Indraprastha Institute of Information Technology, Delhi, New Delhi, India

*To whom correspondence should be addressed. Tel: +91 11 26907438; Fax: +91 11 2690 7405; Email: arjun@iiitd.ac.in
† Equal Contribution

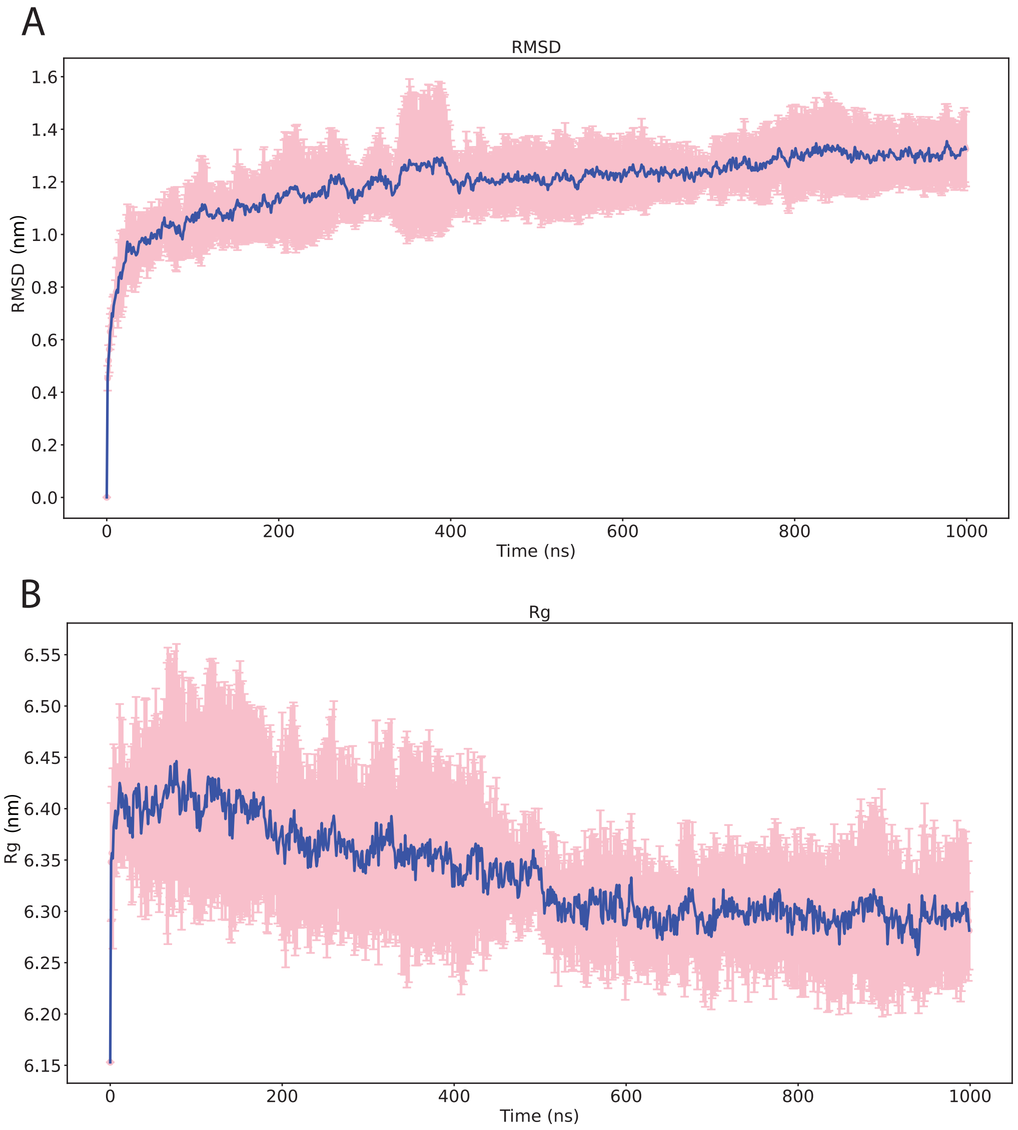

Figure 1: A. Root mean square deviation of backbone of protein across all five simulations. The blue line represents the average while error bars represent the mean absolute deviation observed along simulation time. B. Radius of gyration of protein across all five simulations where blue line represents the average and error bars depict mean absolute deviation.

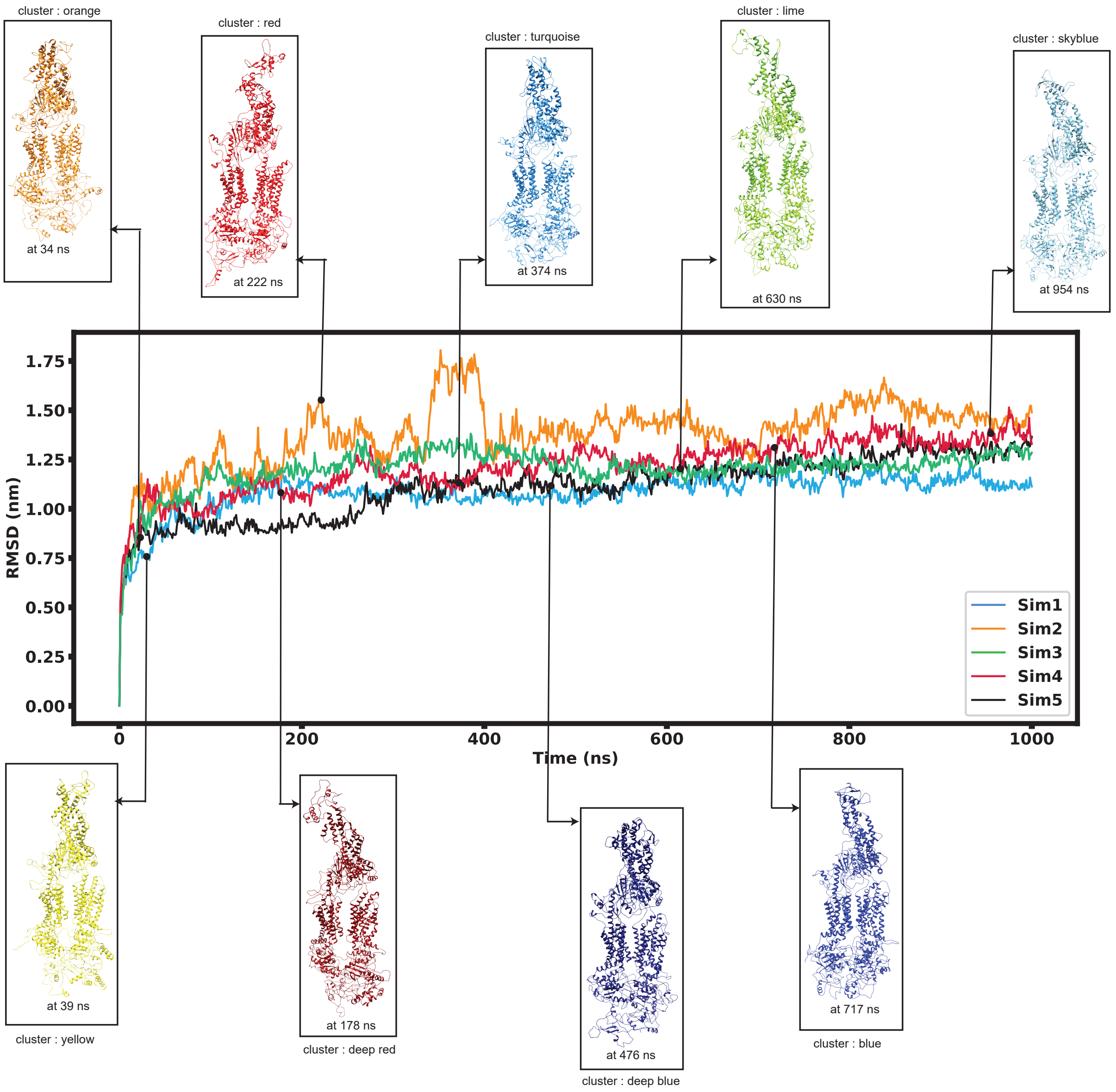

Figure 2: Root mean square deviation of backbone of protein for all the five replicate simulations. Nine conformations of the protein chosen from each cluster bin based on its free energy estimate have been depicted.

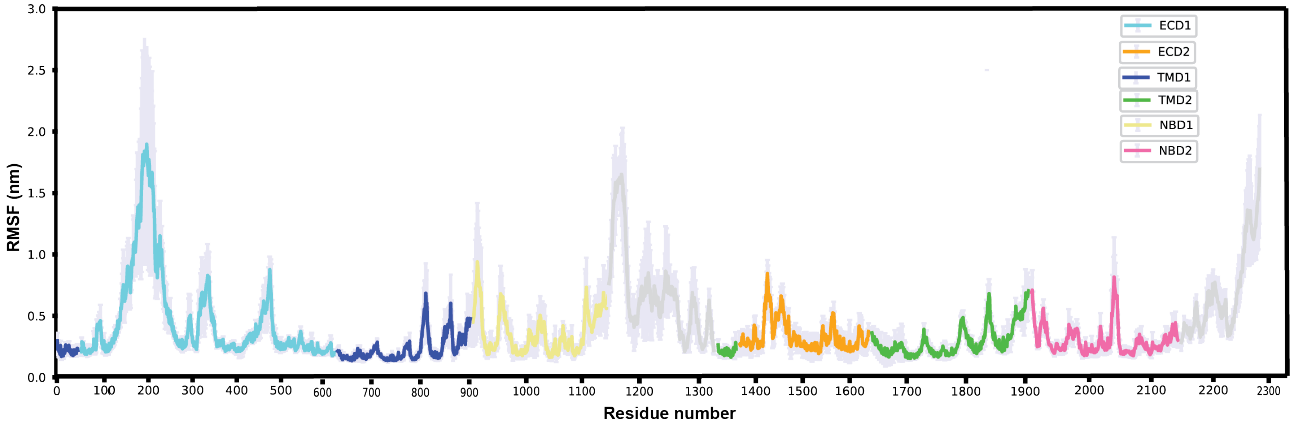

Figure 3: Residue-wise fluctuation (RMSF) observed in all the five simulations. ECD1, ECD2, TMD1, TMD2, NBD1, NBD2 (as designated in Qian et al., 2017) are represented by light blue, orange, navy blue, green, yellow and pink respectively. Region colored in gray represents those residues that have not been classified into any of the domains. The error bars represent the mean absolute deviation.

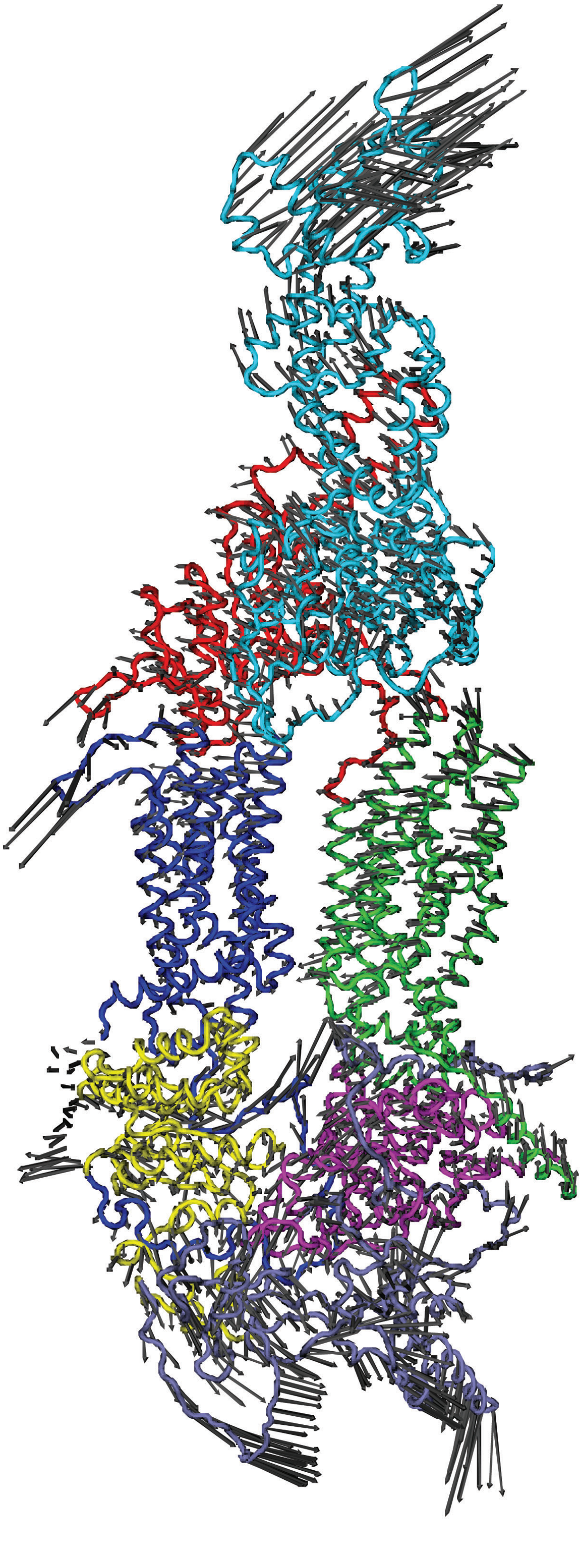

Figure 4: Domain concerted motion of ABCA1. Principal component analysis showing the dominant mode of motion of different domains of ABCA1 throughout the simulation. The direction of arrows (gray) depict the direction of motion of the domain, while their length depicts the intensity. The six domains that make up the protein include ECD1(cyan), ECD2(red), TMD1(blue), TMD2(green), NBD1(yellow), NBD2(magenta) and unspecified region located intracellularly shown in grey color.

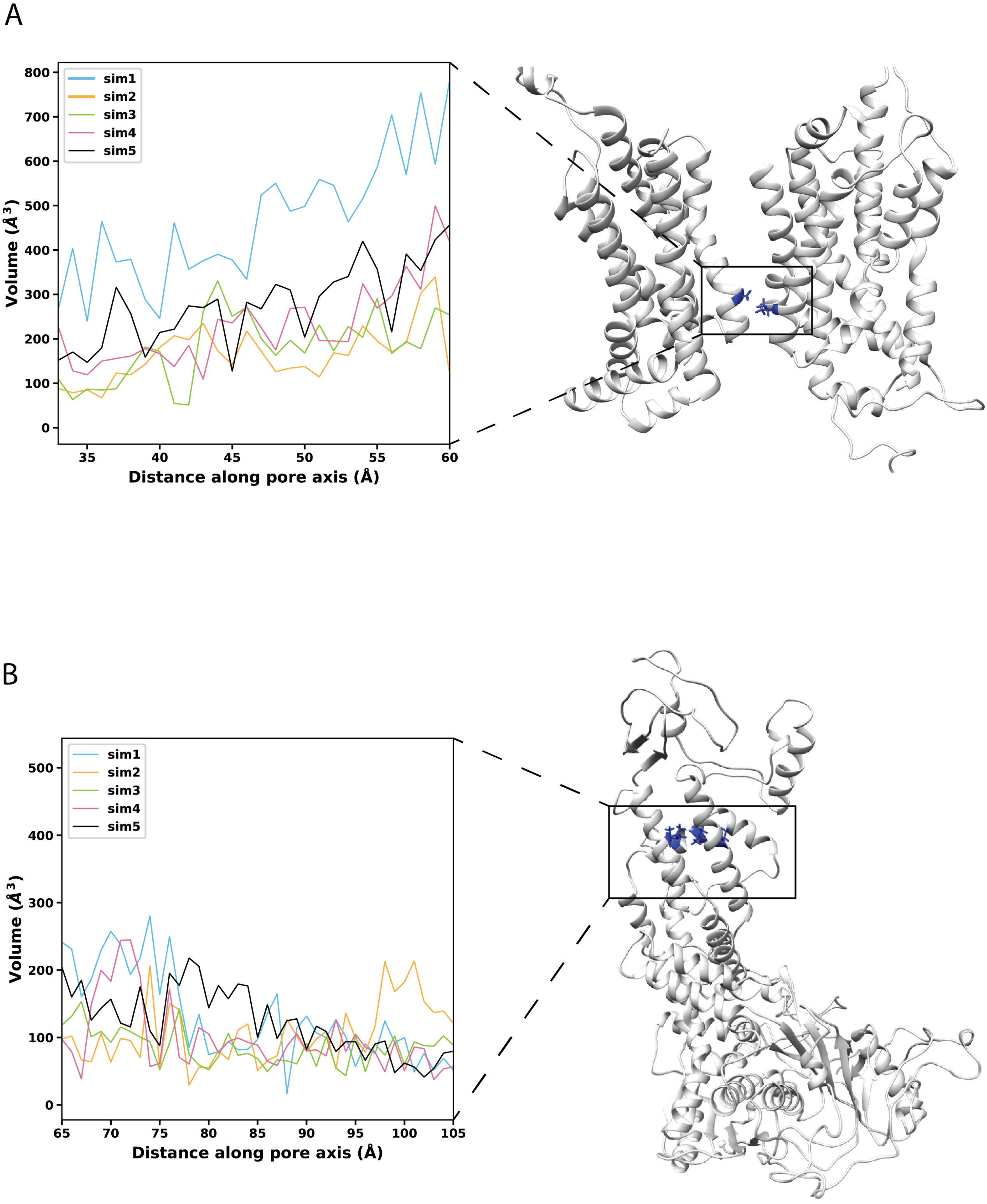

Figure 5: Section of volume profile of tunnel within ABCA1. A. Section of TMDs along with the volume profile of the region where the two TMDs contact each other. B. Section of ECDs along with the volume profile of the distal end of the ECD tunnel. The profiles depict significant narrowing in all the simulations (except one).

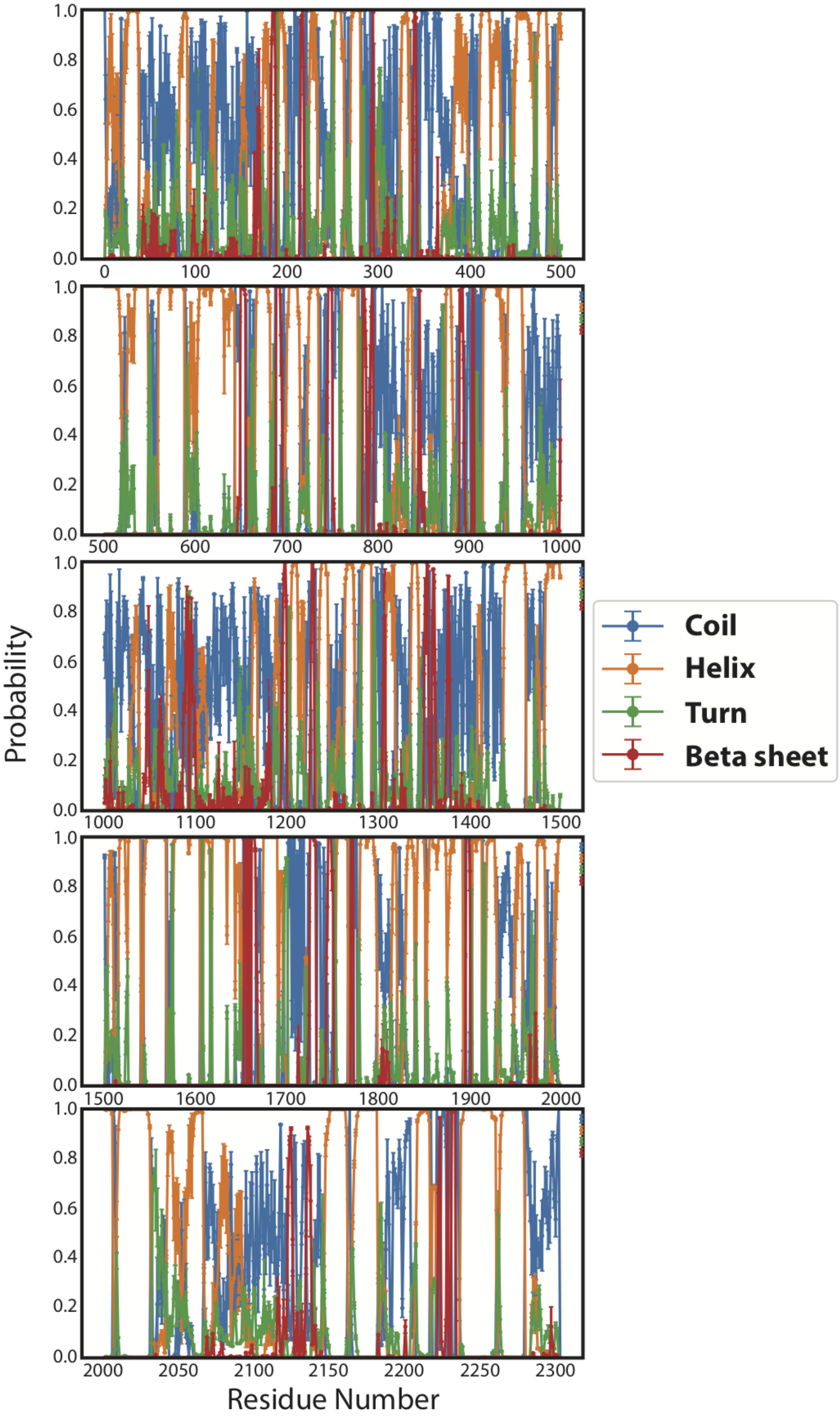

Figure 6: Propensity of each residue of the protein to conform to a particular secondary structure.

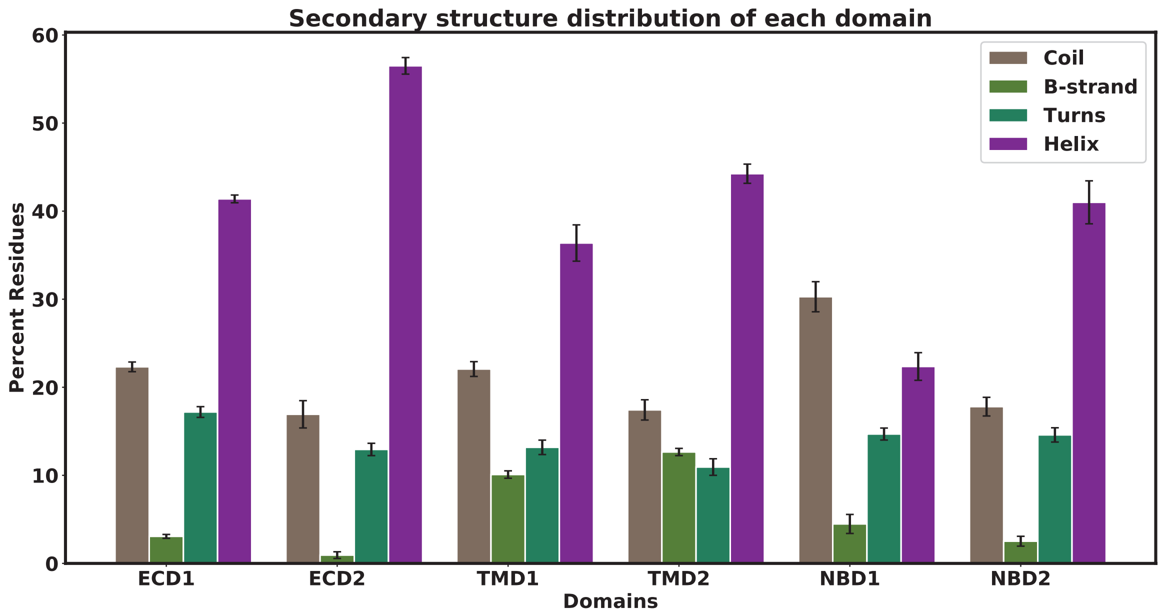

Figure 7: Secondary structure distribution for each domain of ABCA1.

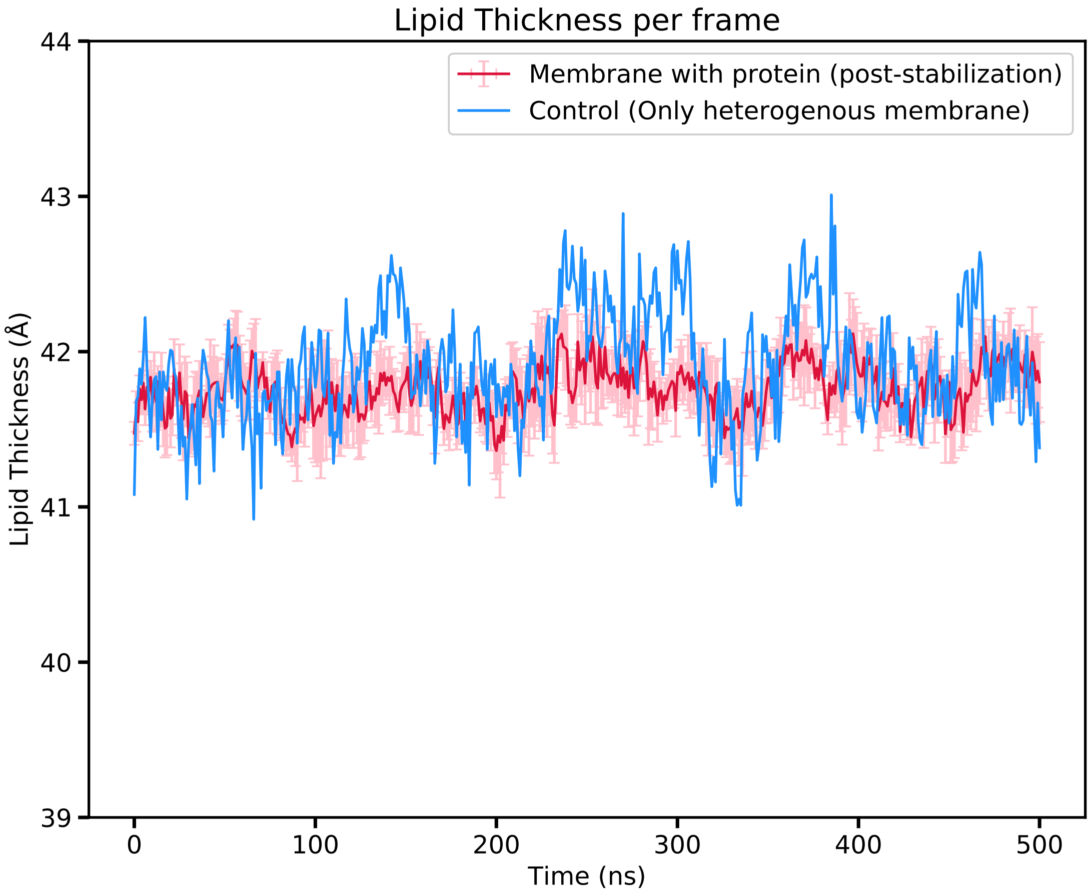

Figure 8: Membrane thickness across the simulation time. Thickness averaged across the top and bottom leaflet has been plotted for the control membrane (blue) as well as membrane with ABCA1 embedded in it (red). Error bars depict the mean absolute deviation in thickness across all five simulations.

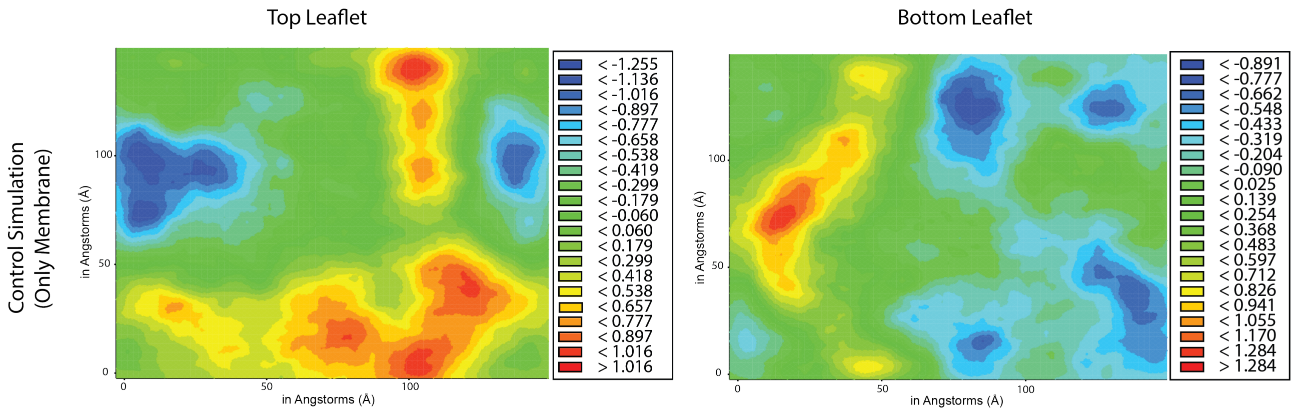

Figure 9: Average thickness map of the top leaflet (left) as well as bottom leaflet (right) for the control membrane.

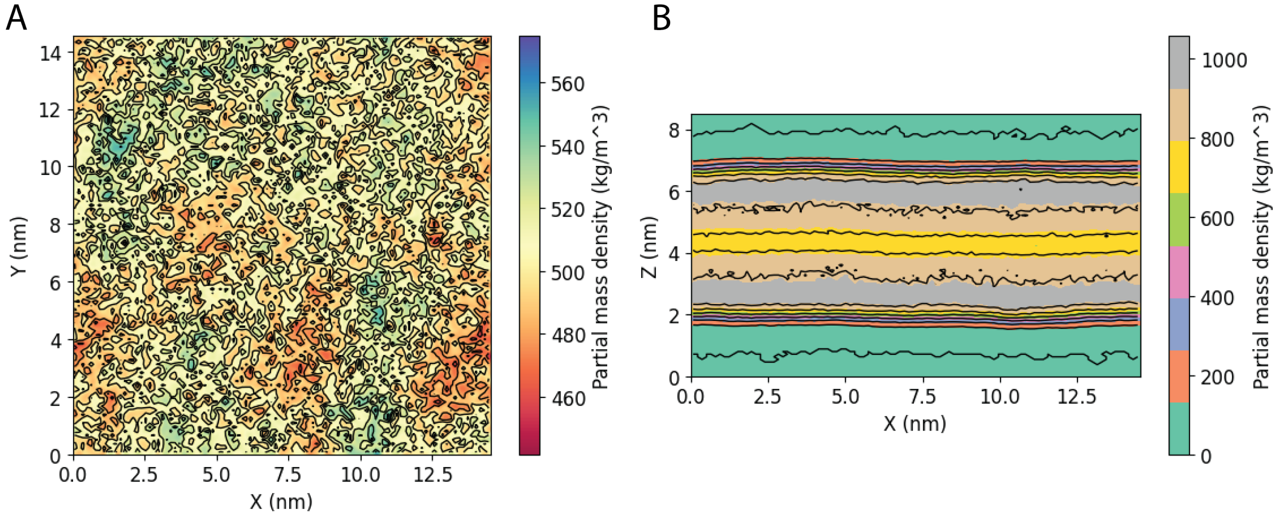

Figure 10: Partial density profile of the control membrane computed along A. XY-plane and B. XZ-plane.

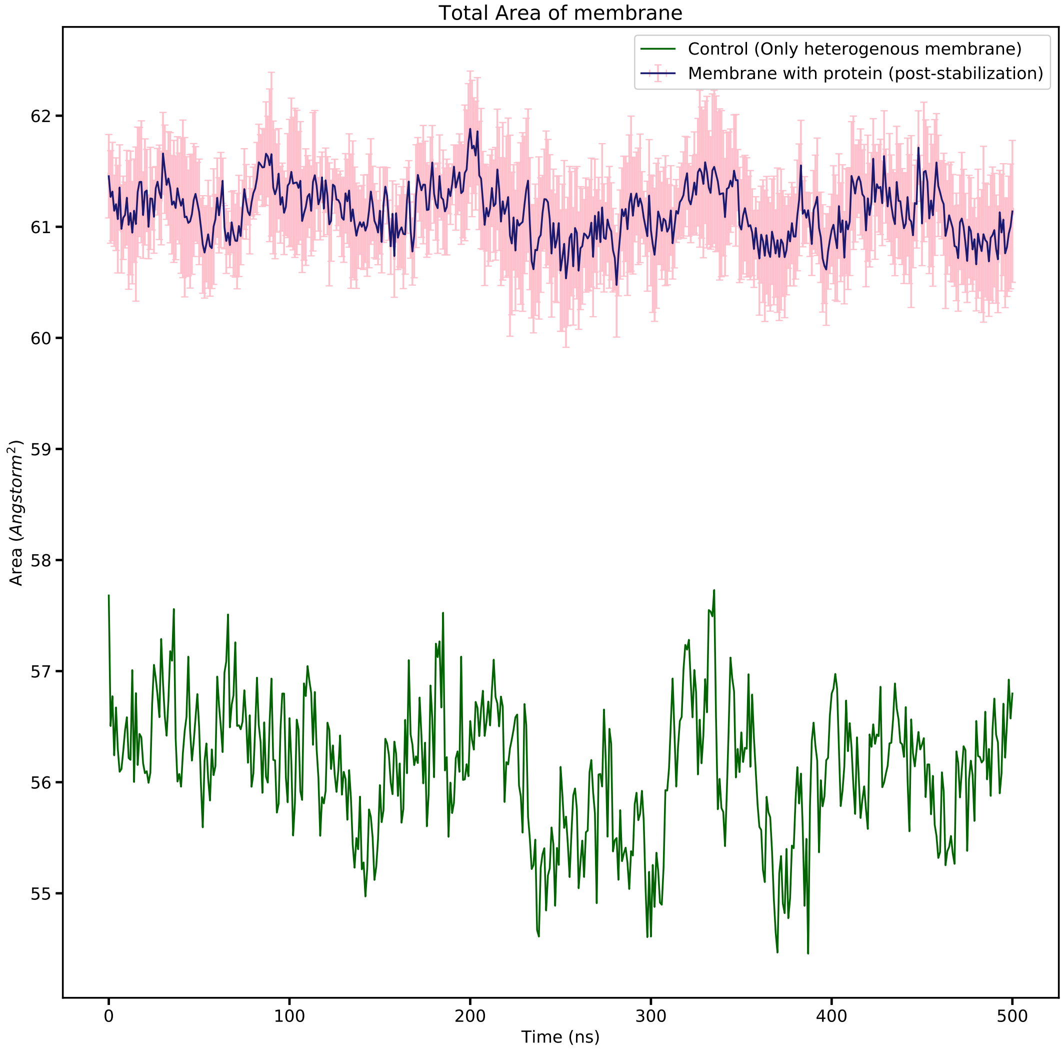

Figure 11: Total area averaged across the top and bottom leaflet for control membrane (green) as well as the membrane with ABCA1 embedded in it (blue). Error bars represent the mean absolute deviation in total area across all five simulations.

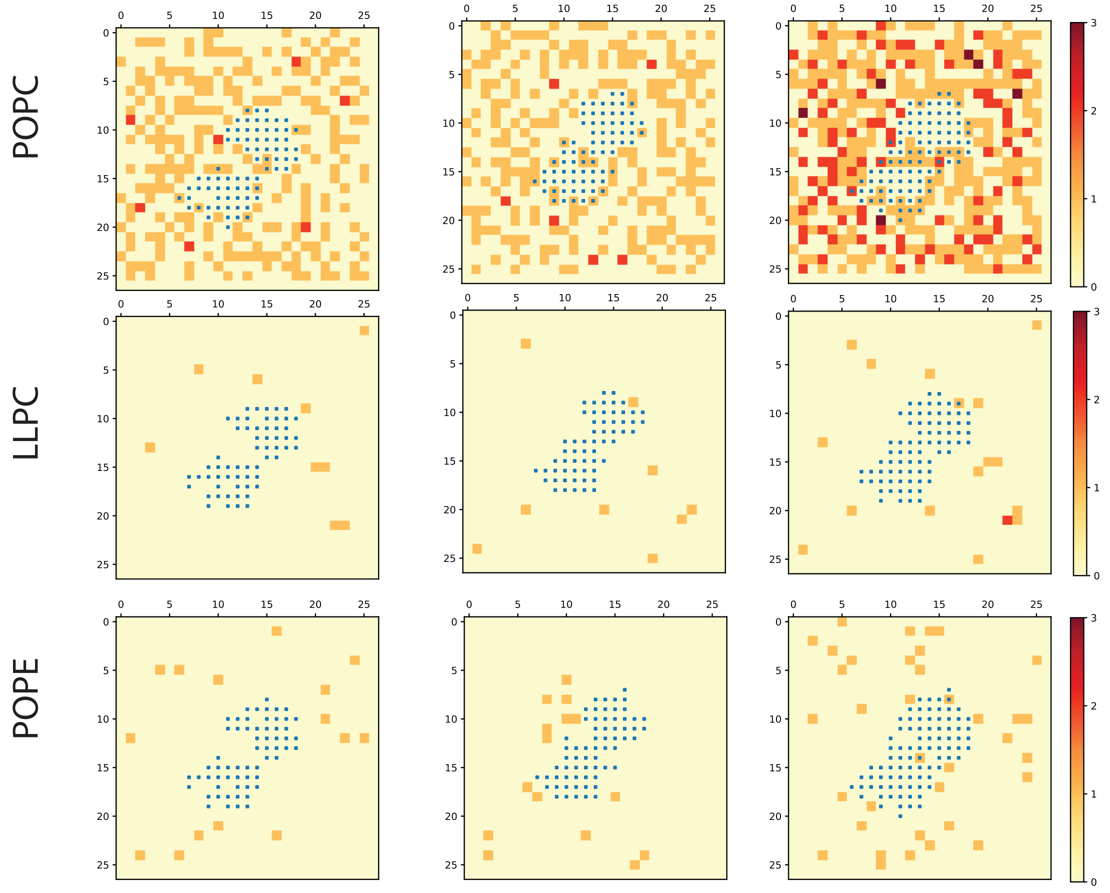

Figure 12: Lipid clustering. Lipids (POPC, LLPC, POPE) colored either as red, orange or cream depending on their frequency around protein (blue squares).The frequency for each lipid was computed for both the top and bottom layer separately and have been shown along with the combined map that represents the total frequency across both leaflets. The membrane is divided into square box of edge 6 Å each and frequency in each of these boxes has been computed and depicted in the figure

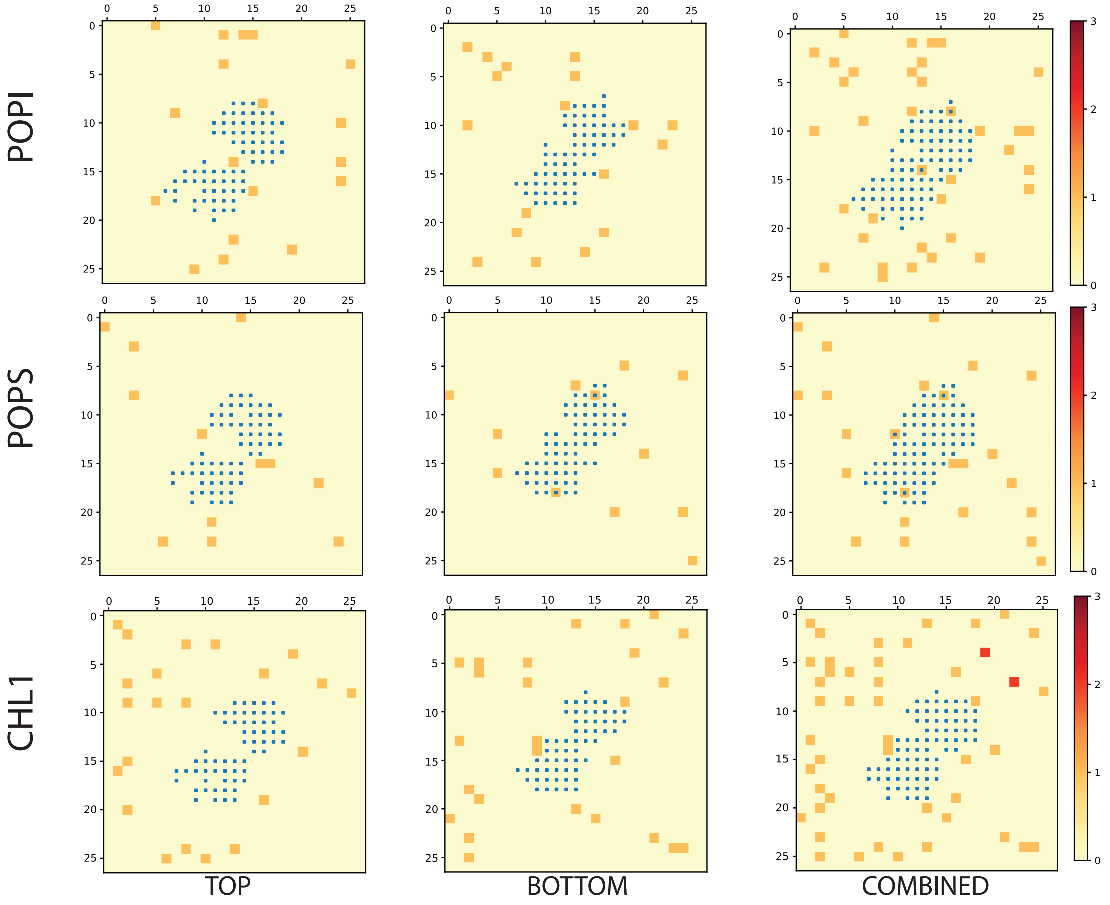

Figure 13: Lipid clustering. Lipids (POPI, POPS, CHL1) colored either as red, orange or cream depending on their frequency around protein (blue squares). The frequency for each lipid was computed for both the top and bottom layer separately and have been shown along with the combined map that represents the total frequency across both leaflets. The membrane is divided into square box of edge 6 Å each and frequency in each of these boxes has been computed and depicted in the figure

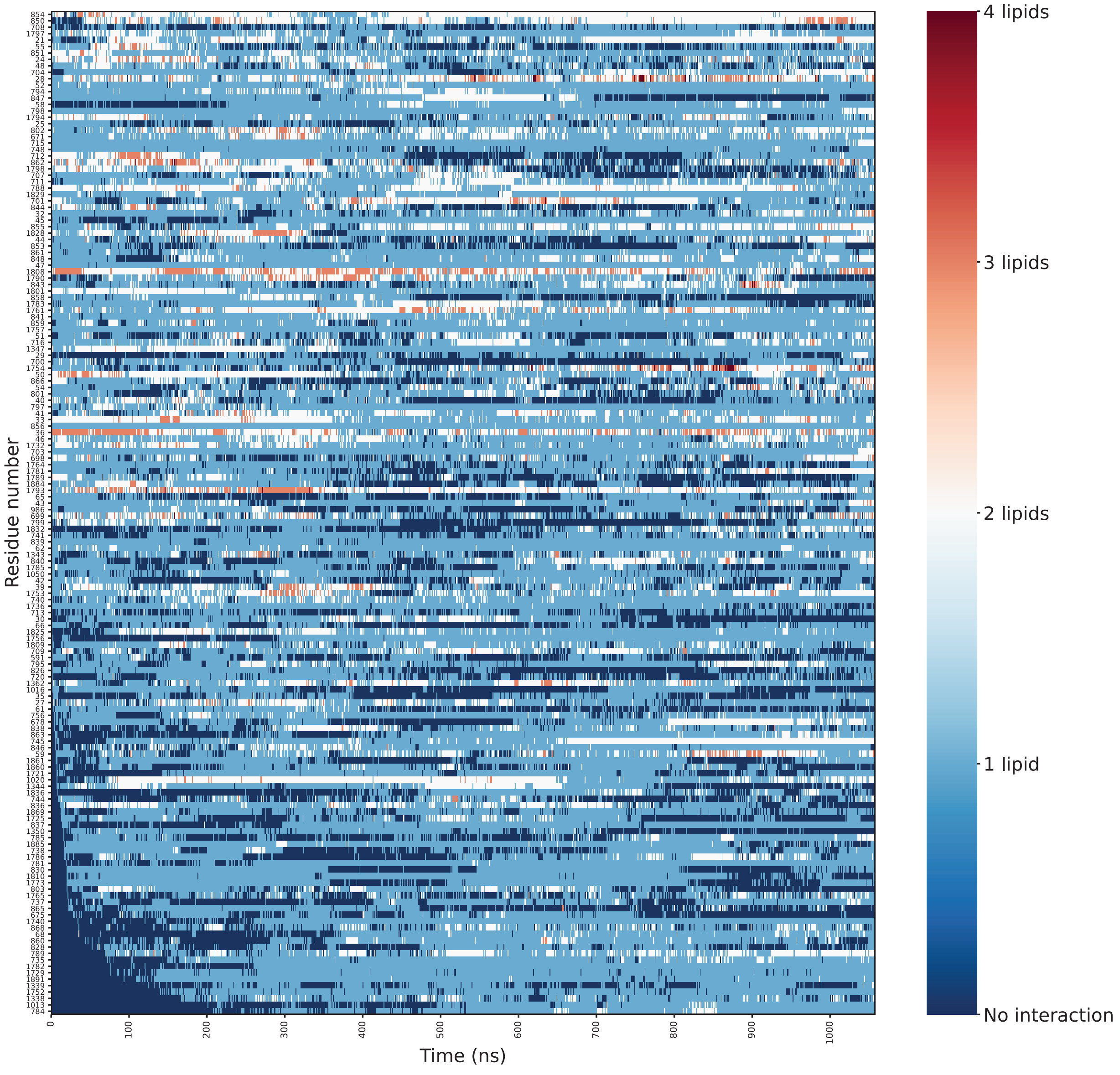

Figure 14: Interactions with lipids (0 or more) throughout the simulation for the 156 protein residues (those that stayed in contact with lipids for >500 ns of simulation time).

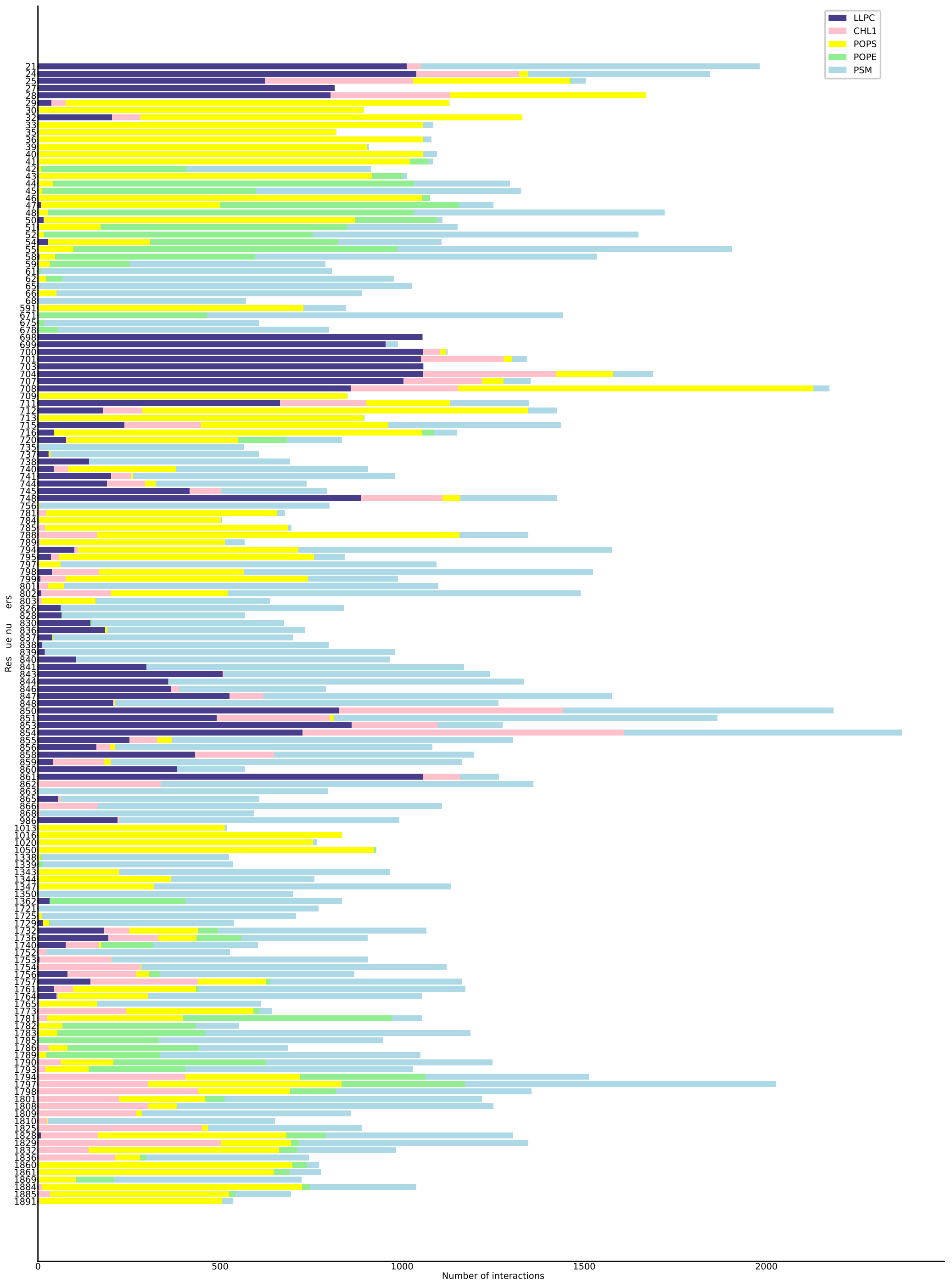

Figure 15: Number of interactions between protein residues in contact with lipids. Frequency of interaction (lipid-wise) for those protein residues that remained in contact with lipids for at least half the simulation time (500ns) has been depicted. POPC is not shown in the figure as its total number of interactions with protein residues was observed to mask all the others.
